# Is an all‐phase ITV (internal target volume) a gold standard in the target definition of hepatocellular carcinoma?

**DOI:** 10.1002/acm2.14532

**Published:** 2024-09-16

**Authors:** Rishabh Kumar, Anil Gupta, Bhaskar Vishwanathan, Rose Kamal, Deepak Thaper

**Affiliations:** ^1^ Department of Radiation Oncology Amrita School of Medicine Faridabad Haryana India; ^2^ Department of Radiation Oncology Subharti Institute of Cancer Management and Research Meerut Uttar Pradesh India; ^3^ Present address: Subharti Medical College Meerut Uttar Pradesh India

**Keywords:** four‐dimensional computed tomography, hepatocellular carcinoma, image‐guided, liver neoplasms, organ motion, radiotherapy

## Abstract

**Background:**

Stereotactic ablative body radiation (SABR) is a well‐recognized treatment option for hepatocellular carcinoma (HCC). Due to the inherent motion of liver tumors, effective motion management is crucial for successful SABR. In the motion‐encompassing motion management technique, all 10 respiratory phase image datasets are delineated and designated as the internal target volume (ITV). Some treatment centers use single or combination image sets to delineate the target volume. This study determines which specialty image set most closely matches an all‐phase ITV contour on a synchronized contrast‐enhanced 4DCT.

**Materials and methods:**

Synchronized 4DCT contrast and delayed scans were acquired for 10 patients in the study. The maximum intensity projection (MiP), average intensity projection (AvgIP), and minimum intensity projection (MinIP) images were generated. The ITV delineation was done in all 10 phases (ITV_all_phase). The ITV_2phase combines the peak inhale and exhale phase, ITV_2 M combines MiP and MinIP, and ITV_3 M combines MiP, MinIP, and AvgIP. All ITVs were compared to ITV_all_phase with Dice similarity index (DSI) and volumes.

**Results:**

Using ITV_all_phase as the reference, the DSI and the mean ITV volumes for the different ITVs were as follows: ITV_all_phase (1 and 116.69 cc), ITV_2phase (0.87 and 105.27 cc), MiP (0.76 and 98.24 cc), AvgIP (0.72 and 94.54 cc), ITV_MinIP (0.67 and 81.08 cc), ITV_2 M (0.84 and 106.26 cc), and ITV_3 M (0.86 and 112.51 cc).

**Conclusion:**

The study demonstrates that in the motion‐encompassing technique of motion management, the target volume generated by delineating all phases of 4DCT provides the most accurate representation for patients with HCC. Specialty image sets and their combinations, while sometimes close, tend to result in less accurate targeting. Hence, the all‐phase 4DCT method should be preferred to avoid geographical misses and ensure optimal treatment outcomes. However, our conclusion may be limited by the technique we employed.

## 1 INTRODUCTION

Stereotactic ablative body radiotherapy (SABR) is now an increasingly used treatment for unresectable hepatocellular carcinoma (HCC).^1^ However, delivering SABR in HCC poses three key challenges: one, the tumor motion, characterized by nonlinear dynamics and hysteresis^2^; second, the challenge of distinguishing between tumor and adjacent healthy liver tissue due to similar computed tomography (CT) densities; and last, the radiosensitivity of the surrounding healthy liver tissue.

Addressing the first challenge involves employing breath‐hold simulation scans or 4D‐CT simulation scans when patients cannot sustain a breath hold. The second challenge is tackled by utilizing two contrast phase simulation planning CT scans, leveraging HCC's distinct radiological characteristics, such as arterial enhancement and delayed washout phases. Simultaneously addressing both challenges requires synchronized contrast‐enhanced 4DCT simulation scans with two contrast phases.

We have developed a contrast timing formula that facilitates synchronized contrast‐enhanced 4DCT simulation for treatment planning, capturing arterial enhancement, and delayed washout phases. This approach yields diagnostic‐quality images essential for accurately delineating the volume of HCC tumors.^3^

The third key challenge can only be addressed by accurately delineating the moving tumor volume. When employing a motion‐encompassing technique, the standard approach is delineating the tumor across all respiratory phase images in the dataset. However, variations exist among centers in the use of different combinations and specialty image datasets for tumor delineation. For instance, maximum intensity projection (MiP) or minimum intensity projection (MinIP) images are often utilized, emphasizing arterial phase enhancement and delayed phase washout characteristics.^4^ There is no clarity on the choice of the specialty image set to contour the target volume in HCC.

This study aims to determine which specialty image set is the closest to an all‐phase gross target volume (GTV) contour on a synchronized contrast‐enhanced 4DCT.

## 2 MATERIAL AND METHODS

### 2.1 Patient selection

This retrospective review was approved by our internal review board, and informed consent was taken from all the patients who were enrolled. The study examined a total of 10 patients who had undergone SABR treatment from May 2018 to January 2020 in the Department of Radiation Oncology. In this study, the following criteria were met before enrolment: unresectable hepatocellular carcinoma patients not suitable for radiofrequency ablation (RFA) and breath‐hold, tumor visible on CT, patients with a regular breathing pattern and no allergy to contrast, gave informed consent and with normal liver volume more than 700 cc with ECOG (Eastern Co‐operative Oncology Group) performance status of 0 to 2. The SABR was not offered or deferred in patients who had a total bilirubin level of more than 3 mg/dL, child score B‐8 or higher, ECOG higher than 2, acute viral hepatitis, platelets less than 50 000, AST/ALT levels of more than five times the normal upper limit, PT/INR > 2.2, albumin < 2.8 g/dL, and previous liver‐directed radiation. The SABR was delivered 4 weeks after TACE and 7 days after stopping sorafenib.

### 2.2 4DCT simulation

After ensuring a normal kidney function (ascertained by creatinine levels, 1.3 mg/dL for men and 1.1 mg/dL for women) and a regular breathing pattern using the ANZAI respiratory gating system AZ‐733 V (Anzai Medical Co. Ltd, Tokyo, Japan), the patients were placed in the treatment position using a Vac‐Lok (CIVCO RT, Orange City, Iowa). The ANZAI system uses optical tracking or pressure sensors to generate a breathing pattern. Patients were trained to create a nonerratic uniform breathing pattern 2–4 days before CT simulation. Once the breathing pattern was uniform, defined by regular peaks and troughs of the sine wave breathing cycle, the patients were taken up for the planning CT. A minimum of 16 to 18G IV cannula ensured an adequate contrast flow. The synchronized 4DCT was taken as per our department protocol published elsewhere.^3^ Briefly, two 4DCT scans were acquired, one in the arterial and the other in the delayed phase. The delay for the arterial phase‐contrast CT was calculated individually for each patient, and the delayed phase CT was taken after 5 min. The images were acquired on a GE Discovery PET/CT 710 with time‐of‐flight (TOF PET‐CT) technology having 128 slices GE optima CT equipment (GE Medical Systems, USA). An automatic contrast injector Ulrich CT motion contrast injector (Ulrich medical interface; Ulrich GmbH & Co. KG, Germany) was connected to the IV line, and 125 mL of Visipaque (iodixanol) containing 270 mg of iodine per ml was injected at a rate of 3 mL/s. The image dataset was transferred to the trial version of MIM Maestro (Version 5.1; MIMvista Corp., Cleveland, Ohio, USA). The MiP, average intensity projection (Avg IP), and Min IP were generated.

### 2.3 ITV delineation

We used guidelines provided by Radiation Therapy Oncology Group Consensus Guidelines to identify our target volume.^5^ A predetermined window width/level‐ 146/84 was used to contour all cases.^6^ A single radiation oncologist contoured all the cases (RK), and another radiation oncologist with expertise in GI Oncology confirmed the contours (AG).

Only the parts that showed enhancement on the arterial phase and washout on the delayed phase were included in the target.^7^ The following ITV iterations were contoured using the enhancing and the delayed 4DCT images.

1. ITV__all‐phase_ → The ITV derived by contouring all 10 phases of the phase sorted 4DCT was termed ITV__all‐phase_. The GTV___0 (0 phase) target contour was first confirmed by both the radiation oncologists and then subsequently copied to the next phase. Minor adjustments were made in the subsequent phases, reducing the intraobserver variation. The ITV__all‐phase_ was our gold standard, and the contours on the specialty image sets were matched to the all‐phase ITV.
2. ITV__2P_ → GTV delineation was done on the peak exhale phase (0% respiratory data set) and the peak inhale phase (50% respiratory dataset). After combining these volumes, an ITV__2P_ was generated.
3. ITV__MIP_ → The GTV delineated only on the MiP image dataset.
4. ITV__AvgIP_ → The GTV delineated only on Avg IP image dataset.
5. ITV__MinIP_ → GTV was delineated only on MinIP image dataset.
6. ITV__2 M_ → the ITV__MIP_ was combined with ITV__Min IP._
7. ITV__3 M_ → the ITV__MIP_, ITV__MinIP and_ ITV__Avg IP_ were combined.

**FIGURE 1:**
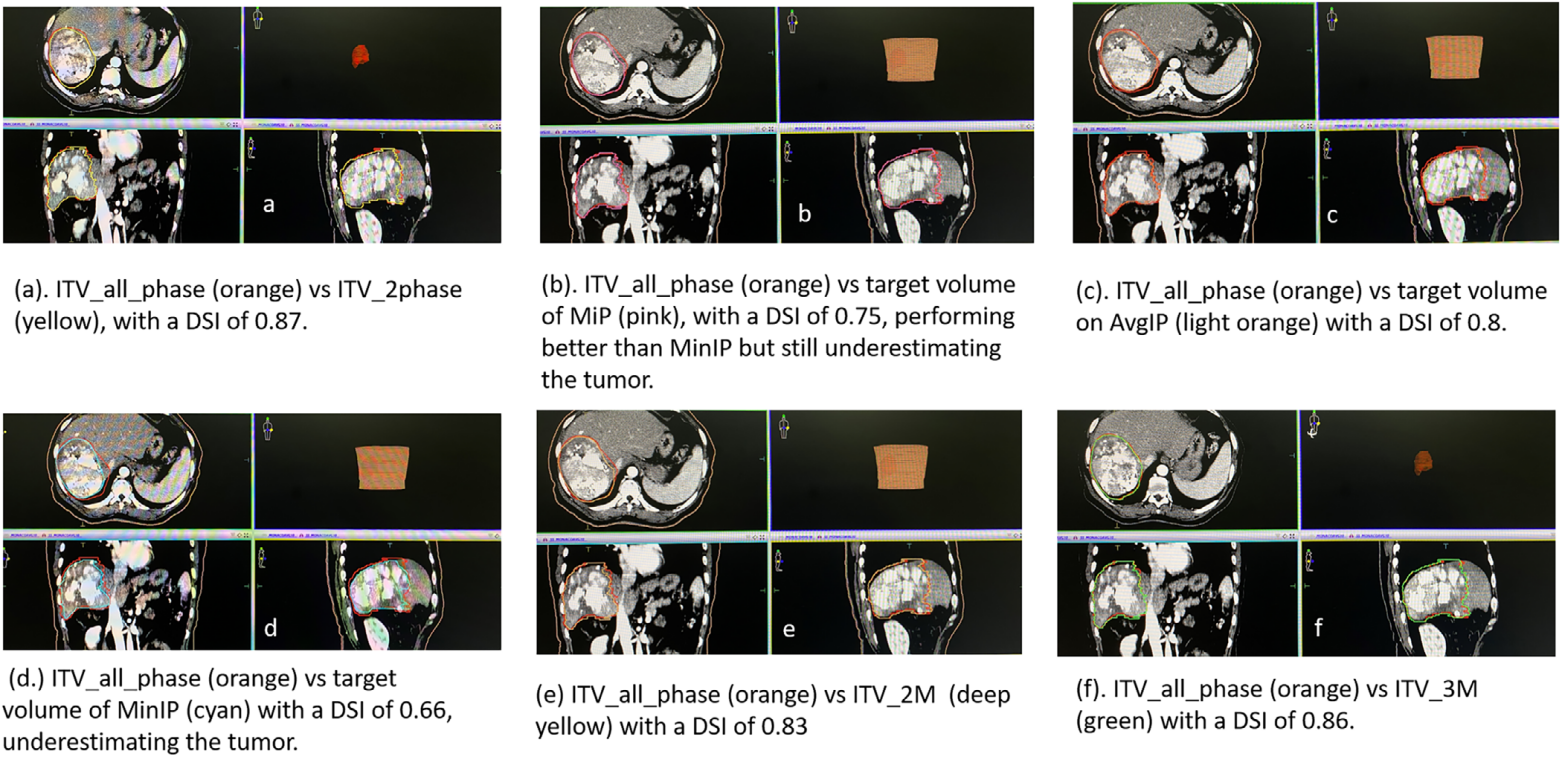
Image showing all the contoured ITVs compared to ITV__all_phase_.

Figure 1 illustrates all the ITV iterations for a single subject. All the ITVs were compared to the ITV__all_phase_ for Dice similarity index (DSI) and target volume. The DSI measures the spatial overlap between two segmentations, A and B target regions, and is defined as DSI (A, B) = 2(A∩B)/(A+B), where ∩ is the intersection. The value of a DSI is a scalar coefficient ranging from 0 to 1, with 0 indicating no spatial overlap between two sets of binary segmentation results and indicating complete or 100% overlap.^8^, ^9^

### 2.4 Target motion

The target motion was calculated in the MiM software in the craniocaudal, anteroposterior, and supero‐inferior directions taking the CT center as the reference.

### 2.5 Treatment planning and delivery

A 5 mm PTV was given to the ITV__all_phase_ for planning. All cases were planned on the delayed 4DCT images, though the dosimetric effect of contrast using our protocol is minimal.^10^ For SABR, all patients were planned using volumetric arc technique (VMAT),^11^ the technical details of the treatment plan, constraints, and clinical details are published elsewhere.^12^, ^13^

### 2.6 Statistical methods

The data was entered, and the measures of central tendency were calculated using SPSS version 23. Wilcoxon signed‐rank test was used to compare tumor volumes and DSI of the various ITVs.

## 3 RESULTS

All patients were classified as BCLC‐C or T4bN0M0 according to the AJCC 8th edition. Three patients had previously undergone TACE (transarterial chemoembolization) treatment. All patients were referred for SABR by the tumor board and were unable to perform breath‐hold, necessitating motion‐encompassing SABR.

The synchronized 4DCT simulation revealed liver motion in all six degrees of freedom. The greatest motion was observed in the superoinferior direction, with a mean of 1.2 cm and a standard deviation of 0.3 cm. Mean motions in the anteroposterior and lateral directions were 0.5  and 0.4 cm, respectively, with a standard deviation of 0.15 cm. Detailed patient‐specific tumor motion characteristics are provided in Table 1.

**TABLE 1:** Tumor motion of liver in patients.

Patient	Liver motion (Superoinferior)	Liver motion (Anteroposterior)	Liver motion (Mediolateral)
1	1.9	0.7	0.6
2	1.2	0.6	0.4
3	1.1	0.2	0.3
4	1.3	0.4	0.4
5	1.2	0.3	0.2
6	1.2	0.5	0.3
7	1.2	0.4	0.4
8	1.2	0.5	0.3
9	1.2	0.6	0.5
10	1.5	0.6	0.4
Mean	1.2	0.5	0.4
Std Deviation	0.3	0.15	0.1

Comparing various ITV sets to ITV_all_phase as the reference, the DSI values were noted as follows: ITV_2phase (0.87), MiP (0.76), AvgIP (0.72), MinIP (0.67), ITV_2 M (0.84), and ITV_3 M (0.86). Poor DSI was observed when a single‐specialty image set was used for ITV generation, with MinIP performing the worst (DSI of 0.67). Further DSI details are outlined in Table 2.

**TABLE 2:** Dice similarity index of all phase ITV (internal target volume) compared to other ITVs.

Dice coefficient	ITV__all‐phase_	ITV__2P_	ITV__MiP_	ITV__AvgIP_	ITV__MinIP_	ITV_2 M	ITV_3 M
Mean	1	0.87	0.76	0.72	0.67	0.84	0.86
Std Dev	0	0.08	0.13	0.16	0.19	0.06	0.05

Mean ITV volumes were measured as follows: 116.69 cc for ITV_all_phase, 105.27 cc for ITV_2phase, 105.27 cc for MiP, 98.24 cc for MinIP, 94.54 cc for AvgIP, 81.08 cc for ITV_2 M, and 112.5 cc for ITV_3 M. The Wilcoxon signed‐rank test indicated significant differences (*p* ≤ 0.005) between the mean volumes of all specialty image sets and ITV_all_phase, except for ITV_3 M (*p* = 0.23). Detailed volume results are provided in Table 3.

**TABLE 3:** Internal target volumes (ITVs) for various combinations.

	ITV__all‐phase_	ITV__2P_	ITV__MiP_	ITV__AvgIP_	ITV__MinIP_	ITV_2 M	ITV_3 M
Patient	Vol (cc)	Vol (cc)	Vol (cc)	Vol (cc)	Vol (cc)	Vol (cc)	Vol (cc)
1	83.2	59.6	16.3	27.5	29.2	35.8	46.5
2	63.5	51.2	38.8	44.9	29.6	44.7	53.8
3	18.3	14.5	9.2	12.3	14.1	15.9	16.2
4	5	3.4	3.9	2.6	1.5	4.3	4.3
5	13.5	9.5	4.3	5.5	2.6	5.6	7.8
6	4.7	3.1	2.9	2.3	1	3.4	4
7	12.9	9.9	7.2	6.7	4.9	8.8	10.1
8	5.8	3.7	3.7	1.3	0.2	4.1	4.2
9	122	106.8	110.1	121.3	87.7	122	128.2
10	838	791	786	721	640	818	850
Median	15.9	12.2	8.2	9.5	9.5	12.35	13.15
Mean	116.69	105.27	98.24	94.54	81.08	106.26	112.51
SD	243.45	230.84	231.38	211.70	188.02	239.75	248.57

## 4 DISCUSSION

For tumors with homogeneous hyperdensity or hypodensity compared to the surrounding normal liver, MiP or MinIP projections should, respectively, reflect the tumor's trajectory across all time‐resolved datasets. MiP images are generated by displaying the highest attenuation values from the dataset, while MinIP images are obtained by displaying the lowest attenuation values.^14^

As a routine practice, most centers use non‐contrast 4D‐CT simulation and fuse it with multi‐phase 3D CT or MRI. Alternatively, they use delayed 4D‐CT, employing Beddar et al.’s delayed‐only synchronized 4D‐CT method.^15^ In those cases, MinIP contour comes closer to ITV generated by contouring all phases, as identified by Lin et al.^4^

However, in our study, most of the tumors were advanced and exhibited heterogeneous density. Using only MinIP underrepresented the target volume, as indicated by a DSI of 0.72. We also employed arterial synchronized 4D‐CT, where the tumor appeared hyperdense due to contrast enhancement. Therefore, combining MinIP with MiP (ITV_2 M) provided a better representation. Nevertheless, our results indicated that even this combination underrepresented the tumor volume, with a DSI of 0.84.

There is a paucity of data on the optimal choice of specialty image sets for ITV generation in patients with HCC, especially when two 4DCTs (arterial and delayed) are acquired. Liver metastases, typically hypodense, are well‐suited for ITV generation using MinIP, which projects the minimum intensity in the CT dataset. HCC, however, shows variability in CT density, becoming hyperdense in the arterial phase and hypodense in the delayed phase. This variability makes using MiP or MinIP in isolation suboptimal. When using arterially enhanced scans to generate MiP, a disadvantage arises because the liver parenchyma does not become sufficiently hypodense to create a clear contrast between the tumor and normal liver, compromising the accuracy of MiP.^14^

As an experimental approach, we combined the AvgIP image dataset with MinIP and MiP (ITV__3 M_), which is not commonly practiced. Our study showed that the DSI for the ITV__3 M_ contour closely approximated the ITV__all_phase_, and there was no significant difference in volume (*p* = 0.23). This is likely because ITV__3 M_ incorporated all CT densities, effectively capturing the mixed‐density nature of HCC. It was also noted that combining two or more specialty datasets increased similarity to ITV__all_phase_ but remained inferior to it. Our results differ from those of Liu et al., who reported that combined MinIP and MiP could be an excellent alternative to the all‐phase ITV for liver tumors. Our DSI was 0.84 compared to their 0.92; this is probably due to using a different synchronized contrast protocol and the fact that they studied only 4 HCC patients.

This technical study is limited by its retrospective nature and small sample size. However, we implemented a rigorous synchronized 4DCT contrast protocol that yielded high‐quality datasets rivalling diagnostic triphasic contrast CT.^6^ The inclusion of both arterial and delayed 4DCT images enabled accurate target delineation; visually inspecting the ITV__all_phase_ video loop confirmed that no tumors were missed. This underscores the importance of contouring across all phases of 4DCT in treating HCC effectively.

## 5 CONCLUSION

Our study suggests that all‐phase ITV should remain the gold standard for ITV generation in patients with HCC. Using a single specialty image set may result in a geographical miss. Combined approaches are slightly better but still are not a match to all phase ITV. A larger sample size is required to ascertain whether combining AvgIP, MinIP, and MiP can be a suitable alternative to all phase contouring in patients with HCC. However, our conclusion may be limited by the technique and inclusion criteria we employed. We used synchronized contrast‐enhanced 4DCT simulation, and the tumor was clearly visible in the multiphase CT. Other treatment planning techniques may yield different results.

## AUTHOR CONTRIBUTIONS

**Rishabh Kumar**: Conceptualization; investigation; writing—original draft; writing—review and editing; methodology. **Anil Gupta**: Conceptualization; methodology; investigation; writing—original draft; writing—review and editing; project administration; visualization; resources. **Bhaskar Vishwanathan**: Supervision. **Rose Kamal**: formal analysis and validation. **Deepak Thaper**: Resources; formal analysis; validation.

Statistical analysis is done by Dr Rishabh Kumar.

No external funding source. Clinical study reports and detailed data tables are available.

## CONFLICT OF INTEREST STATEMENT

The authors declare no conflicts of interest.
